# Early Renal Microcirculatory Perfusion Patterns in Sepsis: Associations with SA-AKI Trajectories in the Emergency Department

**DOI:** 10.3390/diagnostics16081153

**Published:** 2026-04-13

**Authors:** Melih Gökçimen, Gülşen Akçay, Bedriye Müge Sönmez

**Affiliations:** 1 Dışkapı Yıldırım Beyazıt Eğitim ve Araştırma Hastanesi, 06110 Ankara, Turkey; melihgokcimen@gmail.com (M.G.); gulakcay@yahoo.com.tr (G.A.); 2 Ankara Etlik City Hospital, 06170 Ankara, Turkey; 3 Dokuz Eylül Üniversitesi Hastanesi, 35330 İzmir, Turkey

**Keywords:** sepsis, sepsis-associated acute kidney injury, renal resistive index, power Doppler ultrasound, emergency department, renal perfusion

## Abstract

**Background/Objectives:** Sepsis-associated acute kidney injury (SA-AKI) involves complex disturbances in renal microcirculation that may precede overt biochemical evidence of renal dysfunction. This study aimed to characterize early renal perfusion patterns during the emergency department (ED) phase of sepsis, as assessed by the renal resistive index (RRI) and the semiquantitative power Doppler ultrasonography score (SPDUS), and to explore their relationship with subsequent SA-AKI trajectories. **Methods:** In this prospective observational study, adult ED patients who met the Sepsis-3 criteria were enrolled. Renal perfusion was evaluated using the RRI and SPDUS at ED admission and repeated at the fourth hour. SA-AKI was classified as transient or non-transient based on renal recovery patterns. Trajectory comparisons were performed to identify early physiological differences. Receiver operating characteristic (ROC) analyses were conducted for descriptive and exploratory assessment of perfusion pattern separation between injury trajectories. **Results:** Fifty-four patients were included, with 35 classified as transient and 19 as non-transient SA-AKI. Patients with non-transient injury exhibited lower baseline SPDUS_0_ grades and higher RRI_0_ values compared with those with transient injury. These differences were evident at ED presentation, prior to the initiation of advanced organ support, and persisted at the fourth hour, with the non-transient group continuing to show lower SPDUS_4_ and higher RRI_4_ values than the transient group. These findings reflect distinct early renal microcirculatory perfusion patterns across SA-AKI trajectories. Sensitivity, specificity, and cut-off values are reported for descriptive and exploratory purposes only and should not be interpreted as validated clinical thresholds. **Conclusions:** Early alterations in renal microcirculatory perfusion are detectable during the ED phase of sepsis and differ between transient and non-transient SA-AKI trajectories. Baseline RRI and SPDUS values provide physiological insight into early renal perfusion abnormalities and evolving microcirculatory dysfunction in sepsis, but should not be interpreted as predictive tools.

## 1. Introduction

Sepsis remains a leading cause of morbidity and mortality worldwide and is one of the most common life-threatening conditions encountered in the emergency department (ED) [1,2,3]. Among sepsis-related organ dysfunctions, sepsis-associated acute kidney injury (SA-AKI) occurs in up to 40–50% of patients and is independently associated with increased short-term mortality, prolonged hospitalization, higher rates of intensive care unit (ICU) admission, and long-term renal sequelae [2,4,5]. Importantly, SA-AKI is not merely a secondary complication but a key determinant of disease severity and outcome [3].

During the initial phase of sepsis in the ED, before obvious biochemical signs of kidney failure appear, there is a window of opportunity to evaluate renal microcirculation and perfusion with bedside renal Doppler ultrasonography [6,7,8,9,10]. More broadly, recent reviews have highlighted the expanding role of point-of-care ultrasonography (POCUS) in AKI assessment, not only for kidney imaging itself but also for bedside evaluation of renal hemodynamics and multi-organ hemodynamic status in critically ill patients [11]. While renal resistive index (RRI) and semiquantitative power Doppler (SPDUS) techniques have shown promise in critically ill populations [12,13,14,15,16], their applicability and clinical relevance in the ED, where diagnostic uncertainty is high and early risk stratification and management decisions are critical, remain unclear [1].

The ED represents a unique and underexplored physiological window in which renal microcirculatory dysfunction may already be present but potentially modifiable before progression to established or persistent kidney injury. Therefore, this prospective observational study aimed to assess whether the RRI and SPDUS measured at ED presentation are associated with subsequent SA-AKI trajectories, with particular focus on distinguishing transient from persistent SA-AKI and exploring short-term changes during ED stay.

## 2. Materials and Methods

### 2.1. Study Design and Setting

This was a single-center, prospective observational study conducted in the ED of a tertiary care hospital between 1 March 2022 and 1 November 2022. Consecutive adult patients (≥18 years) presenting to the ED who fulfilled the Sepsis-3 diagnostic criteria [17] at admission were screened for eligibility. The study was approved by the institutional review board, and written informed consent was obtained from all participants or their legal representatives.

A priori sample size calculation was performed for the primary comparison of baseline RRI_0_ between transient and non-transient SA-AKI groups. Because no prior emergency department–based study had reported an effect estimate for this specific comparison, the calculation was informed by previously published data from critically ill patients showing a large between-group difference in renal resistive index (0.618 ± 0.081 vs. 0.701 ± 0.073; Cohen’s d ≈ 1.04) [13]. To avoid overestimating the expected effect, a more conservative large effect size (Cohen’s d = 0.80) was assumed. Using a two-sided α of 0.05, 80% power, and a 1:1 allocation ratio, the minimum required sample size was estimated as 52 patients.

### 2.2. Study Population

**Figure 1:**
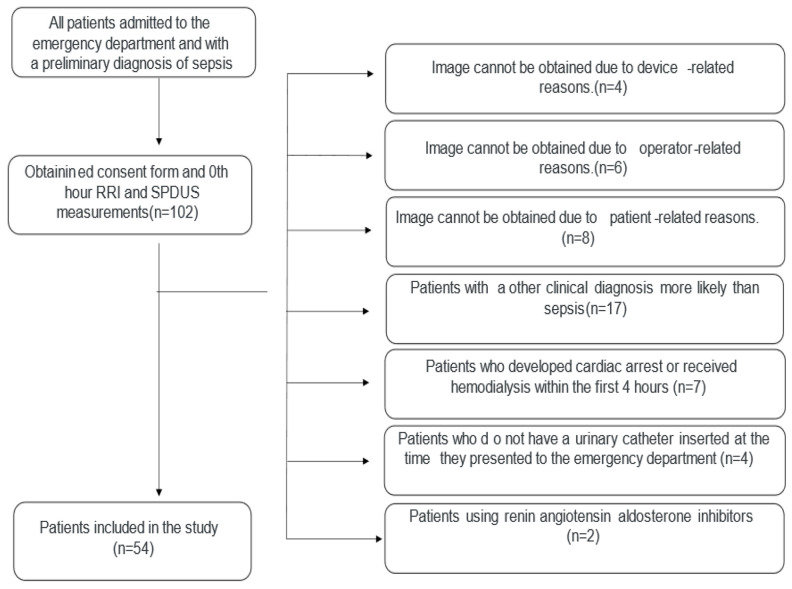
Flow chart of study.

The study included adult patients (≥18 years) who presented to the ED and met Sepsis-3 clinical criteria at admission [17]. Management during ED care was based on contemporary sepsis treatment principles consistent with the 2021 Surviving Sepsis Campaign guidelines [1]. To isolate sepsis-related alterations in renal perfusion and minimize confounding, patients with conditions or treatments known to substantially affect renal blood flow were excluded. Exclusion criteria included pregnancy; trauma; referral from another healthcare facility after initiation of treatment; ED length of stay less than 4 h; known advanced chronic kidney disease (CKD) (documented pre-admission eGFR < 30 mL/min/1.73 m^2^ or known stage 4–5 CKD); diabetes mellitus; essential hypertension; renal artery stenosis; advanced liver failure or hepatorenal syndrome; the use of renin–angiotensin–aldosterone system inhibitors, angiotensin-converting enzyme inhibitors, angiotensin receptor blockers, or diuretics; the absence of urinary catheterization with unknown urine output during the first 4 h; requirement for renal replacement therapy; postrenal acute kidney injury; and cardiac arrest within the first 4 h of ED admission despite successful resuscitation. A flow diagram illustrating patient screening, exclusions, and final enrollment is presented in Figure 1.

### 2.3. Definition of SA-AKI and Renal Recovery Patterns

Acute kidney injury (AKI) was defined according to the Kidney Disease: Improving Global Outcomes (KDIGO) criteria [18]. SA-AKI was operationally defined as AKI occurring in temporal association with sepsis, in the absence of alternative primary renal or postrenal causes [19]. Patients with SA-AKI were classified as having transient or non-transient kidney injury based on renal recovery. Transient SA-AKI was defined as complete normalization of serum creatinine and urine output within 72 h of diagnosis, whereas non-transient SA-AKI was defined as persistence of KDIGO criteria beyond 72 h [20,21]. Serum creatinine was used as the primary parameter for renal function. eGFR was not included in descriptive or comparative analyses because its calculation is unreliable in acute, non–steady-state conditions. Therefore, renal function variables presented in the study reflect acute renal status at presentation rather than confirmed pre-morbid baseline kidney function.

### 2.4. Data Collection

Baseline demographic, clinical, laboratory, and hemodynamic variables were recorded at ED admission using previously prepared data collection forms. These variables included age, sex, known comorbidities, medication history, vital signs at presentation, and mean arterial pressure (MAP), which was specifically recorded as a baseline hemodynamic parameter because systemic hemodynamic status may influence ultrasonographic renal perfusion measurements, along with laboratory tests (including complete blood count, kidney function tests, venous blood gas analysis, and coagulation parameters), urine output, ultrasonographic findings, KDIGO classifications, Sequential Organ Failure Assessment (SOFA), quick Sequential Organ Failure Assessment (qSOFA), Simplified Acute Physiology Score III (SAPS III), source of infection, and clinical outcomes at days 7 and 28.

### 2.5. Ultrasonographic Assessment of Renal Perfusion

Renal ultrasonography was performed to assess early intrarenal perfusion dynamics during the initial phase of sepsis. All examinations were conducted by a single emergency physician with more than four years of experience in emergency ultrasonography and formal certification in basic and advanced ultrasound applications. A single-operator approach was intentionally adopted to ensure internal consistency of image acquisition and measurement technique. The operator was blinded to laboratory results, AKI classification, and subsequent renal outcomes at the time of image acquisition.

All ultrasound examinations were performed using a Mindray M5 ultrasound system (UMT-200 version; Mindray, Mahwah, NJ, USA) with a convex transducer, following standardized acquisition protocols based on the American College of Emergency Physicians renal ultrasound guidelines [10].

Power Doppler ultrasonography was preferred over conventional color Doppler imaging for its superior sensitivity in detecting low-velocity blood flow, particularly within the renal cortex and arcuate vessels [15]. Examples are provided in Supplementary Materials S1–S6.

### 2.6. SPDUS Technique

The order of ultrasonographic measurements was pre-specified in the study protocol. The SPDUS was obtained first to standardize the global assessment of intrarenal perfusion, prior to the repeated vessel targeting, spectral waveform acquisition, and transducer manipulation required for RRI measurement. Because both assessments were completed within the same brief examination window using a fixed sequence, the likelihood of ordering-related bias was considered minimal. SPDUS assessment was performed bilaterally using the same semiquantitative four-grade scoring system. The right kidney was typically examined first for orientation and image acquisition, followed by evaluation of the contralateral kidney using the same technique. Each kidney was initially localized in the transverse plane by scanning between the mid-axillary and anterior axillary lines. After centering the kidney within the acoustic window, the probe was rotated 90 degrees to obtain a coronal view, in which the renal hilum was optimally visualized with minimal rib shadowing. Power Doppler mode was then activated to assess the distribution of intrarenal blood flow. Because the SPDUS grade was identical in both kidneys, a single patient-level SPDUS was recorded for statistical analysis.

All SPDUS images were acquired at end-expiration to reduce respiratory motion artifacts. Renal perfusion was graded using a previously described four-grade semiquantitative scoring system [15]: Grade 0 indicated absence of identifiable intrarenal vessels; Grade 1 indicated visualization of a small number of vessels confined to the hilar region; Grade 2 indicated visualization of hilar and interlobar vessels in most of the renal parenchyma; and Grade 3 indicated visualization of renal vessels extending to the arcuate arteries throughout the entire field of view.

### 2.7. RRI Technique

Following the SPDUS assessment, RRI measurements were obtained from both kidneys using a standardized posterolateral approach. Each kidney was first identified in the transverse plane and subsequently examined in the longitudinal plane. Color Doppler mode was used to identify an interlobar or arcuate artery, after which pulsed-wave Doppler was applied to obtain spectral waveforms.

After visualization of stable waveforms across three consecutive cardiac cycles, peak systolic flow rate (PSFR) and end-diastolic flow rate (EDFR) were measured. The RRI was calculated using the standard formula (PSFR − EDFR)/PSFR. For each kidney, three measurements were obtained from the same arterial level, and the average value was calculated. The mean RRI of both kidneys was used for analysis.

### 2.8. Timing of Measurements and Dynamic Assessment

Baseline ultrasonographic measurements (SPDUS_0_ and RRI_0_) were obtained within the first 6–10 min following ED admission and completed within 5 min to avoid delays in routine care. Follow-up measurements (SPDUS_4_ and RRI_4_) were performed at the 4th hour of ED stay to explore short-term changes in renal perfusion following standard sepsis management in the ED, which included early intravenous fluid resuscitation, administration of appropriate antimicrobial therapy, hemodynamic monitoring, source-directed supportive care, and vasopressor treatment when required according to the patient’s clinical status [1]. After completion of the ED evaluation and treatment, patients were admitted to the ICU or the inpatient ward according to clinical severity and organ support requirements. Although initial ED management followed standard sepsis care principles, subsequent inpatient management was not protocolized by the study and was determined by the treating teams.

### 2.9. Statistical Analysis

Statistical analyses were performed using SPSS Statistics version 28.0 for Windows (IBM Corp., Armonk, NY, USA). Continuous variables were assessed for normality using both the Kolmogorov–Smirnov and Shapiro–Wilk tests. Descriptive statistics were reported as mean ± standard deviation for normally distributed continuous variables, median (interquartile range) for non-normally distributed continuous variables, and n (%) for categorical variables. Because the SPDUS is a four-grade ordinal score, it was summarized as the median (interquartile range) and analyzed using nonparametric methods appropriate for ordinal data. Comparisons between independent groups were performed using the independent-samples *t*-test for normally distributed continuous variables and the Mann–Whitney U test for non-normally distributed variables. Categorical variables were compared using the Chi-square test or Fisher’s exact test, as appropriate. ROC curve analysis was used to describe the ability of baseline renal perfusion parameters to distinguish transient from non-transient kidney injury within the study cohort. Because SPDUS_0_ is a four-grade ordinal score, ROC/AUC analysis for this variable was interpreted within a nonparametric, rank-based framework rather than as an interval-level continuous measure. Accordingly, the AUC for SPDUS_0_ was considered a rank-based summary of its discriminatory ability. The 95% confidence intervals for the AUCs were calculated using DeLong’s nonparametric method. The area under the curve (AUC) was reported as a measure of separation rather than as a means of determining definitive diagnostic cut-off values. To explore the association between early renal perfusion parameters and SA-AKI, logistic regression analyses were performed. Given the study’s physiological focus and limited sample size, multivariable models were intentionally restricted to a small set of clinically relevant covariates to reduce the risk of overfitting. Accordingly, the study was not powered for formal multivariable predictive modeling, and these analyses were considered exploratory and hypothesis-generating. Given the exploratory nature of the between-group comparisons, no formal multiplicity correction was performed. Accordingly, the results, especially borderline *p*-values, should be interpreted cautiously in view of the potential inflation of type I error. All statistical tests were two-sided, and *p*-values < 0.05 were considered statistically significant.

## 3. Results

### 3.1. Demographic and Clinical Characteristics

A total of 54 patients were included in the final analysis, of whom 23 (42.6%) were female and 31 (57.4%) were male. The mean age of the study population was 75.9 ± 14.7 years. Pulmonary infection was the most common source of sepsis (n = 31, 57.4%), followed by genitourinary infections (n = 14, 26.0%). Baseline demographic and clinical characteristics of the study population are summarized in Table 1.

Based on renal recovery patterns during follow-up, 35 patients (64.8%) were classified as transient and 19 patients (35.2%) as non-transient SA-AKI.

### 3.2. Comparison of Transient and Non-Transient SA-AKI

**Figure 2:**
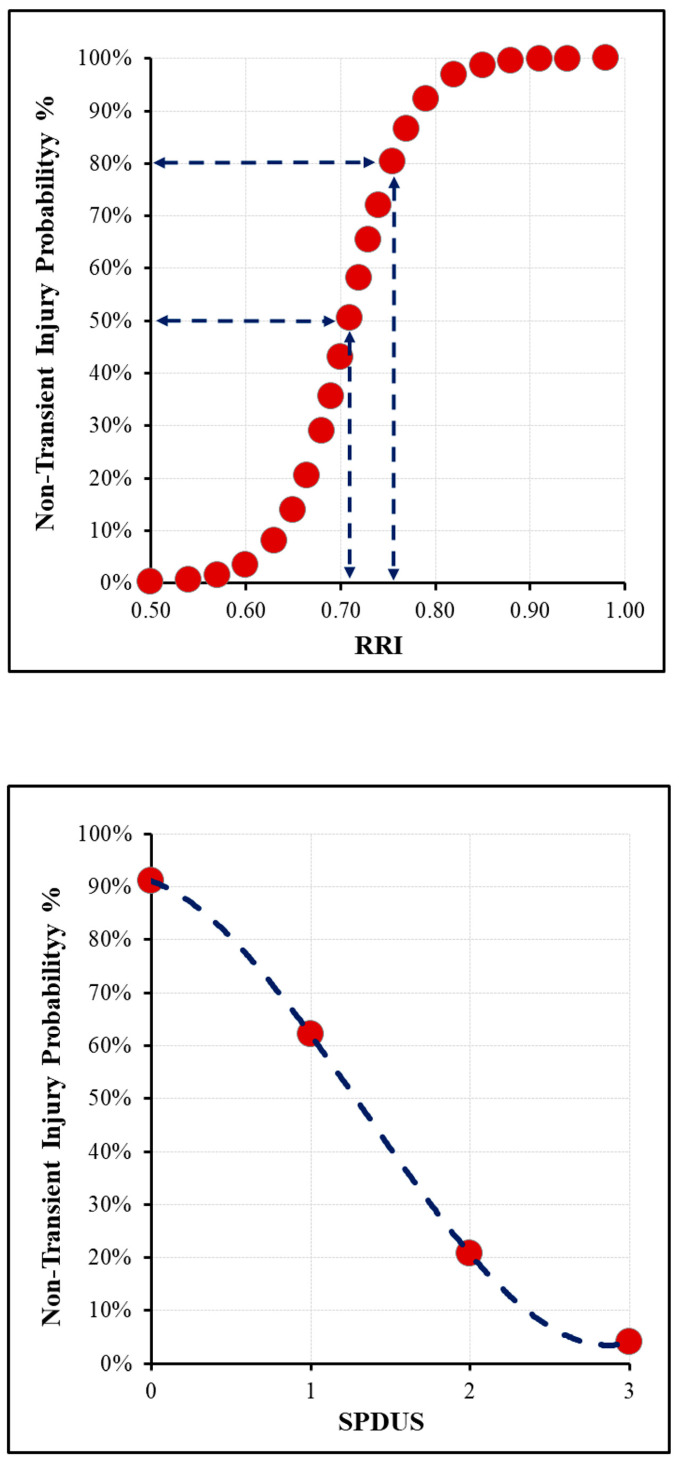
Baseline renal perfusion parameters according to sepsis-associated acute kidney injury (SA-AKI) trajectory.

Comparisons between patients with transient and non-transient SA-AKI are presented in Table 2. Patients with non-transient injury had significantly lower baseline SPDUS_0_ scores and significantly higher baseline RRI_0_ and serum creatinine concentrations than those with transient injury (all *p* < 0.05). The distribution of KDIGO categories at presentation, including patients without AKI at ED admission who subsequently developed SA-AKI during follow-up, did not differ significantly between the transient and non-transient SA-AKI groups (*p* = 0.151). Vasopressor use during ED stay was observed in 9 of 35 patients (25.7%) in the transient SA-AKI group and in 7 of 19 patients (36.8%) in the non-transient SA-AKI group, with no statistically significant between-group difference (*p* = 0.392). In addition, quick Sequential Organ Failure Assessment (qSOFA) and Simplified Acute Physiology Score III (SAPS III) scores were significantly higher in the non-transient injury group, indicating greater overall disease severity. In-hospital mortality was also significantly more frequent among patients with non-transient kidney injury than among those with transient injury (73.7% vs. 37.1%, *p* = 0.010). An inverse relationship was observed between SPDUS_0_ grade and the occurrence of non-transient SA-AKI, whereas higher RRI_0_ values were associated with non-transient SA-AKI, consistent with differing early renal microcirculatory perfusion patterns (Figure 2). At the fourth hour, the non-transient SA-AKI group continued to show lower SPDUS_4_ values and higher RRI_4_ values than the transient SA-AKI group. Median SPDUS_4_ was 3 (2–3) in the transient group and 2 (1–2) in the non-transient group (*p* < 0.001). Mean RRI_4_ was 0.6291 ± 0.0652 in the transient group and 0.7267 ± 0.0430 in the non-transient group (*p* < 0.001). These findings indicate that the between-group perfusion differences observed at ED admission persisted at short-term follow-up during ED stay. These findings are presented in Supplementary Table S2.

### 3.3. Discriminatory Analysis of Baseline Renal Perfusion Parameters

**Figure 3:**
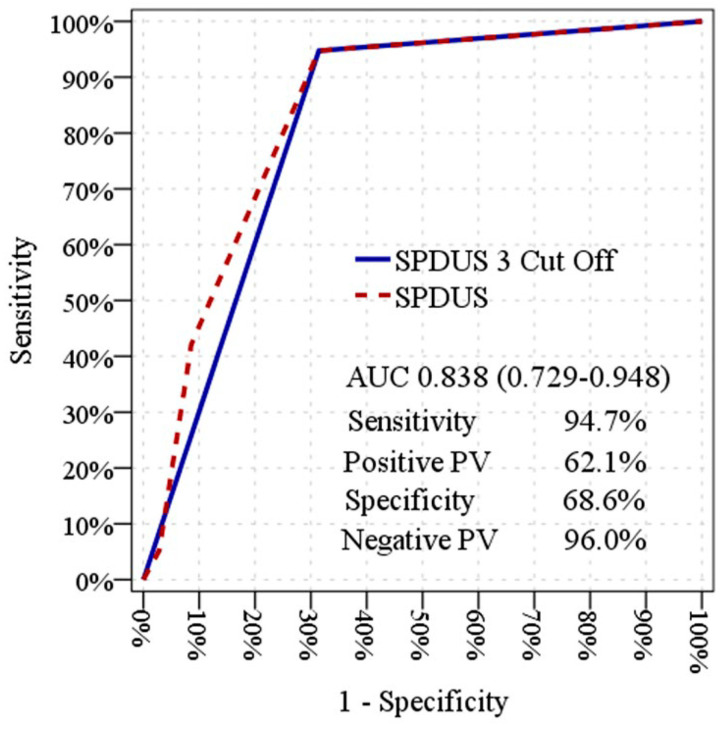
Exploratory receiver operating characteristic (ROC) curve of baseline semiquantitative power Doppler ultrasound score (SPDUS_0_) across transient and non-transient sepsis-associated acute kidney injury.

**Figure 4:**
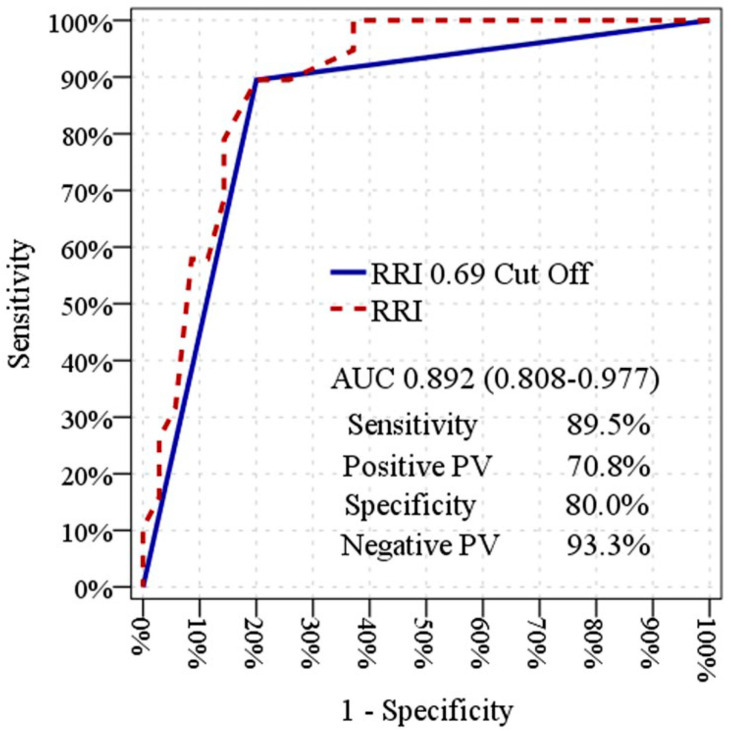
Exploratory receiver operating characteristic (ROC) curve of baseline renal resistive index (RRI_0_) across transient and non-transient sepsis-associated acute kidney injury.

ROC curve analyses were performed to explore the relationship between baseline renal perfusion parameters and SA-AKI trajectories within the study cohort. Baseline SPDUS_0_ demonstrated an area under the ROC curve (AUC) of 0.838 (95% CI: 0.729–0.948; *p* < 0.001). When SPDUS_0_ was evaluated using a grade-based threshold, a similar AUC of 0.817 (95% CI: 0.701–0.932) was observed. Baseline RRI_0_ also showed a high degree of separation between injury trajectories, with an AUC of 0.892 (95% CI: 0.808–0.977; *p* < 0.001) (Table 3; Figure 3 and Figure 4). Sensitivity, specificity, and predictive values associated with SPDUS_0_ and RRI_0_ are reported in Table 3 to describe cohort-specific classification characteristics and should be interpreted as exploratory findings. ROC analyses were performed for descriptive and exploratory purposes to illustrate separation between renal perfusion patterns across injury trajectories rather than to define diagnostic or prognostic thresholds.

### 3.4. Exploratory Multivariable Logistic Regression Analysis

Exploratory multivariable logistic regression analyses were performed to assess whether baseline perfusion parameters remained associated with non-transient SA-AKI after adjustment for illness severity and hemodynamic status (Supplementary Table S1). In models including qSOFA, both baseline RRI_0_ (OR = 1.329, 95% CI: 1.110–1.591; *p* = 0.002) and baseline SPDUS_0_ (OR = 0.236, 95% CI: 0.089–0.627; *p* = 0.004) remained independently associated with non-transient SA-AKI, whereas qSOFA was not independently significant in either model. In additional models including MAP, both RRI_0_ (OR = 1.566, 95% CI: 1.146–2.141; *p* = 0.005) and SPDUS_0_ (OR = 0.136, 95% CI: 0.038–0.478; *p* = 0.002) also remained independently associated with non-transient SA-AKI, while lower MAP was independently associated with non-transient SA-AKI in both models (OR = 0.802, 95% CI: 0.659–0.977; *p* = 0.028; and OR = 0.871, 95% CI: 0.787–0.963; *p* = 0.007, respectively).

## 4. Discussion

This prospective ED-based study showed that early renal microcirculatory perfusion patterns differed between patients who subsequently followed transient and non-transient SA-AKI trajectories. Rather than establishing a predictive or prognostic model, the present study was designed to characterize early intrarenal perfusion differences during the ED phase of sepsis, when conventional biochemical markers may still be insufficient to reflect evolving renal dysfunction.

The principal finding of this study is that baseline renal perfusion parameters were different in patients with transient and non-transient SA-AKI trajectories. This observation supports the concept that renal microcirculatory abnormalities are already detectable early in sepsis, during the ED phase, before prolonged ICU exposure or advanced organ support. In this context, the observed Doppler differences should be interpreted as early physiological pattern differences associated with renal recovery trajectories rather than as evidence of a direct causal or prognostic relationship [2,3,4,5]. Importantly, these differences were not confined to the admission measurements. At the fourth hour of ED stay, the non-transient SA-AKI group continued to exhibit lower SPDUS and higher RRI values, suggesting that the observed differences in perfusion patterns were sustained during short-term follow-up. However, because fourth-hour measurements may also reflect the effects of early therapeutic interventions and evolving hemodynamic changes, these findings should be interpreted as supportive rather than definitive evidence of persistent perfusion abnormalities.

The RRI reflects both renal and systemic hemodynamic influences on intrarenal vascular resistance, whereas SPDUS provides a semiquantitative assessment of cortical and medullary perfusion [14,15,16]. When interpreted together, these measures may provide a more comprehensive physiological description of early renal perfusion status than either parameter alone. Recent reviews have highlighted the growing role of POCUS in evaluating renal hemodynamics and AKI in critically ill patients, supporting the view that Doppler-based renal perfusion assessment may provide clinically relevant bedside physiological information [11]. In this broader context, renal Doppler ultrasound may be clinically useful not only for early monitoring of renal microcirculatory alterations but also for bedside hemodynamic assessment during the initial phase of sepsis. Moreover, integration of renal perfusion findings with other POCUS-based assessments commonly used in septic shock, including cardiac, lung, and venous congestion evaluations, may provide a more comprehensive physiological picture and improve early risk stratification. In line with this physiological interpretation, baseline MAP was significantly lower in the non-transient SA-AKI group. This suggests that the observed differences in baseline the RRI and SPDUS may reflect not only intrarenal microcirculatory alterations but also broader systemic hemodynamic impairment during the early phase of sepsis. However, in the present study, these parameters should be understood as descriptive markers of renal microcirculatory perfusion patterns within this cohort, rather than as validated diagnostic thresholds or prognostic tools for clinical decision-making [22,23].

These findings should also be interpreted in the broader context of early AKI risk stratification. In recent years, biomarker-based approaches, such as urinary [TIMP-2] × [IGFBP7], and functional markers, such as proenkephalin, have been investigated to identify patients at high risk of AKI during the early therapeutic window, particularly in perioperative and critical care settings [24,25]. In parallel, biomarker-guided implementation of supportive measures based on the KDIGO AKI care bundle has been explored as a strategy to reduce progression to clinically significant AKI [26]. In this framework, RRI and SPDUS may occupy a complementary rather than competing role: unlike biochemical markers of kidney stress or dysfunction, they provide a bedside physiological snapshot of renal perfusion and microcirculatory status during the early phase of sepsis [19]. It is plausible that combining perfusion-based ultrasonographic parameters with biochemical biomarkers could add value to early SA-AKI risk stratification, although this hypothesis requires validation in larger prospective studies.

Distinguishing transient from non-transient SA-AKI remains clinically relevant because persistent renal dysfunction has been associated with worse short- and long-term outcomes, including mortality and progression to chronic kidney disease [27,28,29]. Although previous studies have reported mixed findings regarding the role of RRI in differentiating transient from persistent AKI [30], the present results indicate that early Doppler-based perfusion patterns differ between these renal recovery trajectories. These findings support the possibility that the degree of early microcirculatory disturbance is associated with subsequent patterns of renal recovery, although no causal or predictive inferences can be drawn from the present data. In exploratory multivariable models, baseline RRI_0_ and SPDUS_0_ remained associated with non-transient SA-AKI after adjustment for qSOFA and MAP. However, given the limited sample size, these findings should be interpreted as hypothesis-generating rather than as evidence of a definitive independent prognostic effect.

The mortality rate observed in this cohort was high, particularly among patients with non-transient kidney injury. This finding should be interpreted in the context of greater sepsis severity in the non-transient SA-AKI group, as reflected by higher qSOFA and SAPS III scores, together with the advanced age of the cohort and the predominance of ICU admission. SA-AKI has been consistently associated with increased mortality, particularly in elderly patients with limited physiological reserve and multiple comorbidities [2,3,31,32]. Renal recovery itself is an important determinant of outcome, independent of the severity of initial sepsis, underscoring the clinical relevance of distinguishing between persistent and transient kidney injury. The relatively high mean SAPS III score in our cohort, despite a more moderate mean SOFA score, likely reflects the predominance of elderly individuals in the study population and the broader structure of SAPS III, which incorporates age, comorbidity burden, and baseline clinical characteristics in addition to acute physiological derangement.

This study has several important limitations that should be acknowledged when interpreting the findings. First, this was a single-center study with a relatively small sample size, which inherently limits statistical power and external validity. Although the cohort size was sufficient to explore physiological associations, the study was not designed or powered to establish definitive prognostic thresholds or to support broad clinical decision-making. Accordingly, all discriminatory analyses should be interpreted as cohort-specific and hypothesis-generating rather than confirmatory. Although the observed AUC values suggest substantial separation within this cohort, they should not be interpreted as validated predictive thresholds, and external validation in larger multicenter cohorts is required before any broader clinical application can be considered. Second, the study population was highly selected, with extensive exclusion criteria to minimize confounding factors that could affect renal perfusion and Doppler-derived indices. While this approach allowed a more focused assessment of sepsis-related renal microcirculatory alterations, it limits generalizability to the broader, heterogeneous population of critically ill patients with sepsis, in whom comorbid conditions such as chronic kidney disease, hypertension, diabetes mellitus, and vasoactive medication use are common. Consequently, the findings may not be directly applicable to unselected ICU populations, in whom comorbidities and treatment-related confounders are more prevalent. At the same time, this design reflects the clinical reality that patients with sepsis are initially evaluated and managed in the ED, where early physiological alterations precede ICU admission and advanced organ support. From this perspective, the study specifically targets the early diagnostic window during which renal microcirculatory dysfunction may still be modifiable. In particular, the exclusion of diabetes mellitus, essential hypertension, and RAAS inhibitor use was intended to reduce chronic vascular and renal hemodynamic confounding that could influence Doppler-derived perfusion indices, but this came at the cost of reduced external validity. Because these conditions are highly prevalent among patients with sepsis in routine clinical practice, the present findings should be interpreted as applying primarily to a selected population rather than to unselected real-world sepsis cohorts. Available comorbidity data did not suggest that the non-transient group represented a broadly more multimorbid population; rather, between-group differences appeared to be more closely related to acute illness severity within this selected cohort. Third, ultrasonographic measurements were performed by a single experienced operator, and neither interobserver nor intraobserver variability was formally assessed. Although this strategy improved internal consistency and reduced technical variability, it introduces potential operator-dependent bias and limits reproducibility across centers and operators with different levels of ultrasound expertise. In addition, SPDUS is inherently subjective, and despite standardized acquisition protocols, grading variability cannot be fully excluded. Accordingly, the cohort-specific AUC values reported in this study should not be extrapolated to multi-operator settings or assumed to reflect SPDUS performance beyond this single-operator context. Fourth, renal perfusion parameters are influenced by systemic hemodynamics, vascular compliance, and age-related arterial stiffness. Although efforts were made to obtain measurements early, prior to major therapeutic interventions and before vasopressor exposure, residual confounding by factors such as baseline blood pressure, cardiac output, and evolving volume status cannot be excluded. Moreover, because baseline MAP was lower in the non-transient SA-AKI group, it is not possible to fully disentangle the contribution of systemic hemodynamic impairment from that of intrarenal microcirculatory alterations in the observed RRI and SPDUS differences. In addition, vasopressor exposure specifically at the time of fourth-hour ultrasonographic reassessment, as well as dose-related effects, was not recorded separately. Because both vasopressor therapy and fluid resuscitation may influence Doppler-derived renal indices, their potential influence on RRI_4_ cannot be completely excluded. Fifth, pre-admission baseline creatinine was unavailable for 17 of 54 patients (31.5%), which may have introduced misclassification in distinguishing transient from non-transient SA-AKI. In these patients, renal recovery had to be interpreted based on in-hospital creatinine trends and urine output rather than on a confirmed pre-morbid renal baseline. As the group assignment was based on the 72 h clinical course and creatinine trajectory rather than a standardized pre-morbid reference, misclassification of borderline cases cannot be completely excluded. In addition, although trajectory classification was based on 72 h renal recovery, detailed descriptors of creatinine dynamics, such as peak serum creatinine, rate of rise, and time-to-peak, were not reported separately. Therefore, heterogeneity in the magnitude and temporal pattern of kidney injury within each trajectory group may not have been fully captured, potentially introducing bias into the between-group comparisons. Because the transient versus non-transient SA-AKI classification was determined over a 72 h period that extended beyond the ED stay, variation in post-ED management may have influenced renal recovery trajectories. Although urine output was clinically followed and considered during classification of renal recovery, it was not systematically recorded in the final study dataset as a standalone, analyzable variable. Therefore, separate quantitative reporting of urine output findings was not possible. In addition, serum creatinine and urine output—used as reference standards—are imperfect surrogates for early kidney injury and may lag behind microcirculatory dysfunction, further complicating temporal interpretation. Finally, the predominance of elderly individuals in the study population introduces additional confounding due to age-associated vascular changes and reduced renal reserve. Given the strong associations among age, sepsis severity, and mortality, it is not possible to fully disentangle the independent contribution of renal perfusion parameters from the overall disease burden and host vulnerability. Multicenter studies that incorporate larger, more diverse populations and standardized hemodynamic adjustments are required to validate these findings and clarify their clinical relevance.

## 5. Conclusions

In this prospective ED-based study, baseline renal perfusion parameters differed with subsequent renal injury patterns in patients with sepsis. These findings suggest that bedside RRI and SPDUS may provide valuable physiological insight into early sepsis-related alterations in renal microcirculation. Rather than serving as definitive diagnostic or prognostic tools, baseline RRI and SPDUS should be interpreted as descriptors of early perfusion disturbances that may be relevant to subsequent patterns of renal recovery. Larger multicenter studies are required to validate these findings, determine optimal measurement strategies, and define how renal perfusion assessment may complement early risk stratification and monitoring in sepsis. Importantly, these findings are most applicable to the early phase of sepsis management in the ED, where patients are first encountered and where identification of renal microcirculatory dysfunction prior to ICU admission may offer a critical opportunity for risk stratification and timely intervention.

## Figures and Tables

**Table 1:** Baseline Characteristics of Study Population.

Total (n = 54)	
Age, years	75.9 ± 14.7
Sex, n (%)	
Male	31 (57.4)
Female	23 (42.6)
Kidney injury pattern, n (%)	
Transient SA-AKI	35 (64.8)
Non-transient SA-AKI	19 (35.2)
Baseline renal perfusion parameters	
SPDUS	2.22 ± 0.86
Renal resistive index (RRI)	0.67 ± 0.08
Baseline renal function	
Serum creatinine (mg/dL)	2.17 ± 1.41
Severity scores	
SOFA score	4.8 ± 2.9
qSOFA score	1.74 ± 0.76
SAPS III score	78.9 ± 17.6
Source of infection, n (%)	
Pulmonary	31 (57.4)
Genitourinary	14 (26.0)
Gastrointestinal	4 (7.4)
Soft tissue	2 (3.7)
Hepatobiliary	2 (3.7)
Central nervous system	1 (1.9)

Values are presented as mean ± standard deviation or number (%), as appropriate. SPDUS: semiquantitative power Doppler ultrasound score; RRI: renal resistive index; SA-AKI: sepsis-associated acute kidney injury; SOFA: Sequential Organ Failure Assessment; qSOFA: quick SOFA; SAPS III: Simplified Acute Physiology Score III.

**Table 2:** Comparison of transient and non-transient sepsis-associated acute kidney injury.

	Transient SA-AKI (n = 35)	Non-Transient SA-AKI (n = 19)	*p* Value
Age, years	76.0 (66.0–85.5)	84.0 (76.0–85.5)	0.077
MAP	71.88 ± 10.68	59.47 ± 13.81	0.001
SPDUS_0_ score	3.0 (2.0–3.0)	2.0 (1.0–2.0)	<0.001
RRI_0_	0.63 ± 0.07	0.74 ± 0.05	<0.001
Serum creatinine (mg/dL)	1.33 (0.97–2.58)	2.36 (1.61–2.96)	0.031
KDIGO stage [at presentation, n (%)]			0.151
No Injury	10 (30.3%)	4 (26.7%)	
Stage 1	11 (33.3%)	1 (6.7%)	
Stage 2	6 (18.2%)	4 (26.7%)	
Stage 3	6 (18.2%)	6 (40.0%)	
SOFA score	4.0 (3.0–5.0)	5.0 (2.5–8.5)	0.226
qSOFA score	1.0 (1.0–2.0)	2.0 (1.5–3.0)	0.027
SAPS III score	69.0 (60.0–80.3)	88.5 (74.5–100.9)	0.006
Vasopressor use	9 (25.7%)	7 (36.8%)	0.392
ICU admission, n (%)	24 (68.6%)	16 (84.2%)	0.210
In-hospital mortality, n (%)	13 (37.1%)	14 (73.7%)	0.010

Values are presented as mean ± standard deviation for normally distributed continuous variables, median (interquartile range) for non-normally distributed continuous variables or ordinal variables, and n (%) for categorical variables. Group comparisons were performed using the independent-samples *t*-test for normally distributed continuous variables, the Mann–Whitney U test for non-normally distributed continuous or ordinal variables, and Pearson’s chi-square test or Fisher’s exact test for categorical variables, as appropriate according to expected cell counts. SPDUS_0_ indicates semiquantitative power Doppler ultrasound score at admission; RRI_0_, renal resistive index at admission; KDIGO, Kidney Disease: Improving Global Outcomes; SOFA, Sequential Organ Failure Assessment; qSOFA, quick SOFA; SAPS III, Simplified Acute Physiology Score III. KDIGO stage at presentation includes patients without AKI at ED admission (No Injury), who subsequently developed SA-AKI during follow-up.

**Table 3:** Exploratory ROC analysis of baseline renal perfusion parameters for distinguishing transient and non-transient SA-AKI.

	AUC	95% CI	*p* Value
SPDUS_0_ (rank-based ROC analysis)	0.838	0.729–0.948	<0.001
SPDUS_0_ < 3 (cut-off)	0.817	0.701–0.932	<0.001
RRI_0_ (continuous)	0.892	0.808–0.977	<0.001
RRI_0_ ≥ 0.69 (cut-off)	0.847	0.735–0.960	<0.001

Using a cut-off value of SPDUS_0_ < 3, sensitivity was 94.7%, specificity was 68.6%, positive predictive value (PPV) was 62.1%, and negative predictive value (NPV) was 96.0%. Using a cut-off value of RRI_0_ ≥ 0.69, sensitivity was 89.5%, specificity was 80.0%, PPV was 70.8%, and NPV was 93.3%. SPDUS_0_ indicates semiquantitative power Doppler ultrasound score at emergency department admission; RRI_0_, renal resistive index at admission; AUC, area under the receiver operating characteristic curve; CI, confidence interval. Sensitivity, specificity, and cut-off values are reported for descriptive and exploratory purposes only and should not be interpreted as validated clinical thresholds.

## Data Availability

The datasets used and/or analyzed during the present study are available from the corresponding author on reasonable request.

## Supplementary Materials

The following supporting information can be downloaded at: https://www.mdpi.com/article/10.3390/diagnostics16081153/s1, Figure S1: Representative pulsed-wave Doppler measurement obtained from an interlobar renal artery after color Doppler localization. Peak systolic and end-diastolic velocities are shown, and the corresponding renal resistive index (RRI) is displayed on the image. Figure S2: Representative pulsed-wave Doppler tracing from an interlobar renal artery demonstrating repeated waveform sampling for calculation of the renal resistive index (RRI). Figure S3. Representative arcuate artery Doppler measurement showing spectral waveform acquisition and calculation of the renal resistive index (RRI). Figure S4. Representative Doppler image of an arcuate renal artery after color Doppler localization, with corresponding spectral waveform used for renal resistive index (RRI) calculation. Figure S5. Representative interlobar artery spectral Doppler tracing used for calculation of the renal resistive index (RRI). Figure S6. Representative repeated interlobar artery Doppler measurements demonstrating acquisition of spectral waveforms for renal resistive index (RRI) assessment. Table S1. Exploratory logistic regression models evaluating associations between baseline perfusion parameters and non-transient SA-AKI. Table S2. Baseline and fourth-hour renal perfusion parameters according to transient and non-transient sepsis-associated acute kidney injury (SA-AKI).

## Abbreviations

The following abbreviations are used in this manuscript:

AUROC	area under the receiver operating characteristic curve
CD	color Doppler
ED	emergency department
EDFR	end-diastolic flow rate
KDIGO	Kidney Disease: Improving Global Outcomes
MAP	mean arterial pressure
PSFR	peak systolic flow rate
PWD	pulsed wave Doppler
qSOFA	quick Sequential Organ Failure Assessment
RRI	renal resistive index
SA-AKI	sepsis-associated acute kidney injury
SAPS III	Simplified Acute Physiology Score III
SCr	serum creatinine
SOFA	Sequential Organ Failure Assessment
SPDUS	semiquantitative power Doppler ultrasonography score
US	ultrasonography
