# Supplementary material for: Early Renal Microcirculatory Perfusion Patterns in Sepsis: Associations with SA-AKI Trajectories in the Emergency Department
Source: Diagnostics (Basel). 2026 Apr 13;16(8):1153. doi: 10.3390/diagnostics16081153 (PMC13115345; doi:10.3390/diagnostics16081153)
Supplement: Supplementary file 1 [file diagnostics-16-01153-s001.zip › Table S1.pdf]

Table S1. Exploratory logistic regression models evaluating associations between baseline perfusion parameters and non-transient SA-AKI

Model	Variables included	OR	95% CI	p value	Model fit
Model 1	RRI_0	1.329	1.110–1.591	0.002	Omnibus $p<0.001$; Nagelkerke $R^2=0.566$; Hosmer–Lemeshow $p=0.594$
	qSOFA	1.596	0.525–4.851	0.409	
Model 2	$SPDUS_0$	0.236	0.089–0.627	0.004	Omnibus $p=0.001$; Nagelkerke $R^2=0.373$; Hosmer–Lemeshow $p=0.284$
	qSOFA	2.063	0.798–5.332	0.135	
Model 3	RRI_0	1.566	1.146–2.141	0.005	Omnibus $p<0.001$; Nagelkerke $R^2=0.766$; Hosmer–Lemeshow $p=0.972$
	MAP	0.802	0.659–0.977	0.028	
Model 4	$SPDUS_0$	0.136	0.038–0.478	0.002	Omnibus $p<0.001$; Nagelkerke $R^2=0.583$; Hosmer–Lemeshow $p=0.836$
	MAP	0.871	0.787–0.963	0.007	

OR, odds ratio; CI, confidence interval; RRI_0, renal resistive index at admission; $SPDUS_0$, semiquantitative power Doppler ultrasound score at admission; MAP, mean arterial pressure; SA-AKI, sepsis-associated acute kidney injury. Models were intentionally kept parsimonious because of the limited sample size and should be interpreted as exploratory and hypothesis-generating.
