# Supplementary material for: Early Renal Microcirculatory Perfusion Patterns in Sepsis: Associations with SA-AKI Trajectories in the Emergency Department
Source: Diagnostics (Basel). 2026 Apr 13;16(8):1153. doi: 10.3390/diagnostics16081153 (PMC13115345; doi:10.3390/diagnostics16081153)
Supplement: Supplementary file 1 [file diagnostics-16-01153-s001.zip › Table S2.pdf]

Table S2. Baseline and fourth-hour renal perfusion parameters according to transient and non-transient sepsis-associated acute kidney injury (SA-AKI)

	Transient SA-AKI	Non-transient SA-AKI	*p* value
$SPDUS_0$	3 (2–3)	2 (1–2)	<0.001
$SPDUS_4$	3 (2–3)	2 (1–2)	<0.001
RRI_0	0.6321 ± 0.0693	0.7413 ± 0.0513	<0.001
RRI_4	0.6291 ± 0.0652	0.7267 ± 0.0430	<0.001

Values are presented as mean ± standard deviation for normally distributed continuous variables, median (interquartile range) for non-normally distributed continuous variables or ordinal variables, and n (%) for categorical variables. Group comparisons were performed using the independent-samples t-test for normally distributed continuous variables and the Mann-Whitney U test for non-normally distributed continuous or ordinal variables. $SPDUS_0$ and $SPDUS_4$ indicate semiquantitative power Doppler ultrasound scores at admission and at 4 hours, respectively; RRI_0 and RRI_4, renal resistive index at admission and at 4 hours, respectively.
