# Supplementary figures and images for: Early Renal Microcirculatory Perfusion Patterns in Sepsis: Associations with SA-AKI Trajectories in the Emergency Department
Source: Diagnostics (Basel). 2026 Apr 13;16(8):1153. doi: 10.3390/diagnostics16081153 (PMC13115345; doi:10.3390/diagnostics16081153)

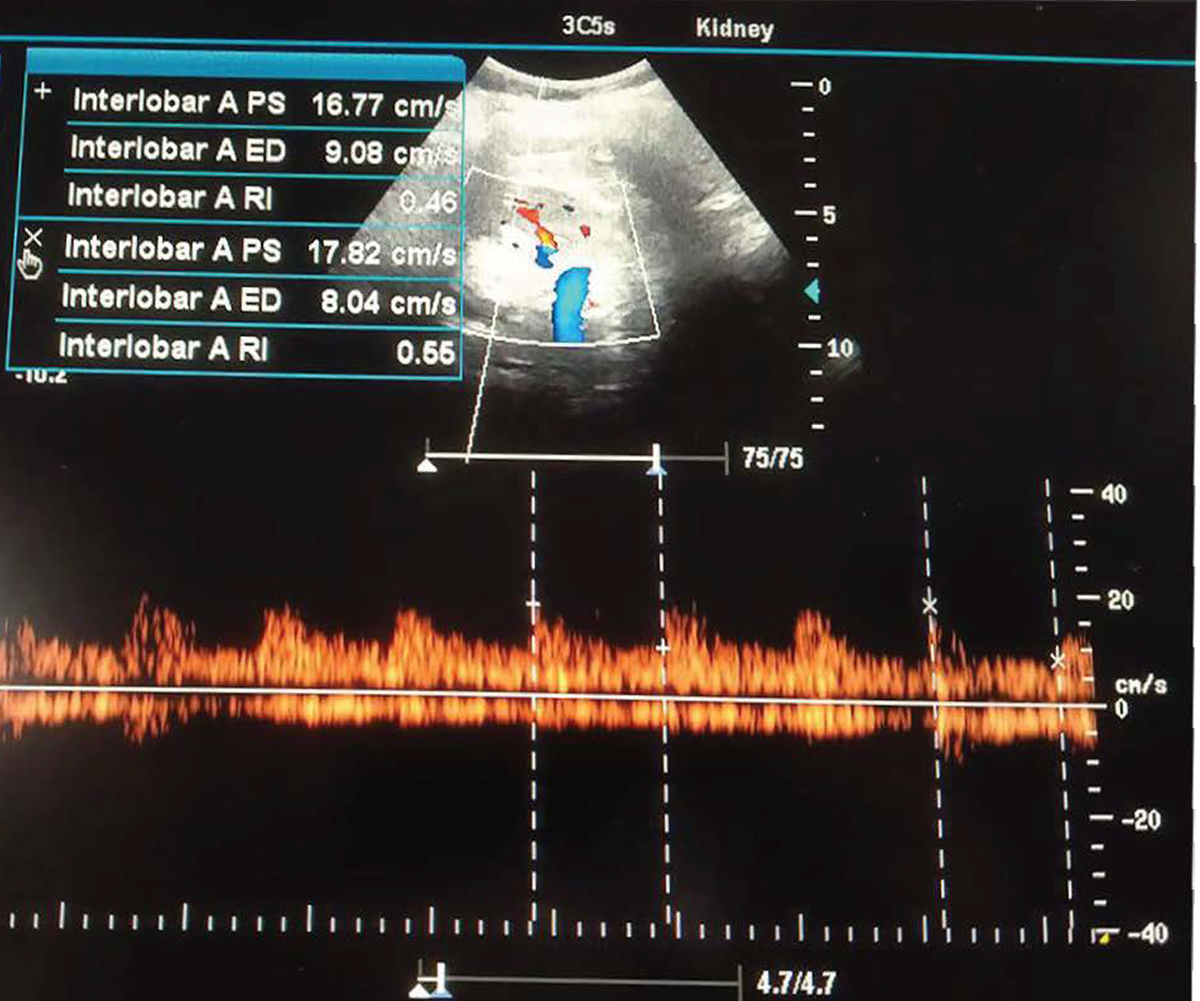

Supplement: Supplementary file 1 [file diagnostics-16-01153-s001.zip › Figure S1.tif]

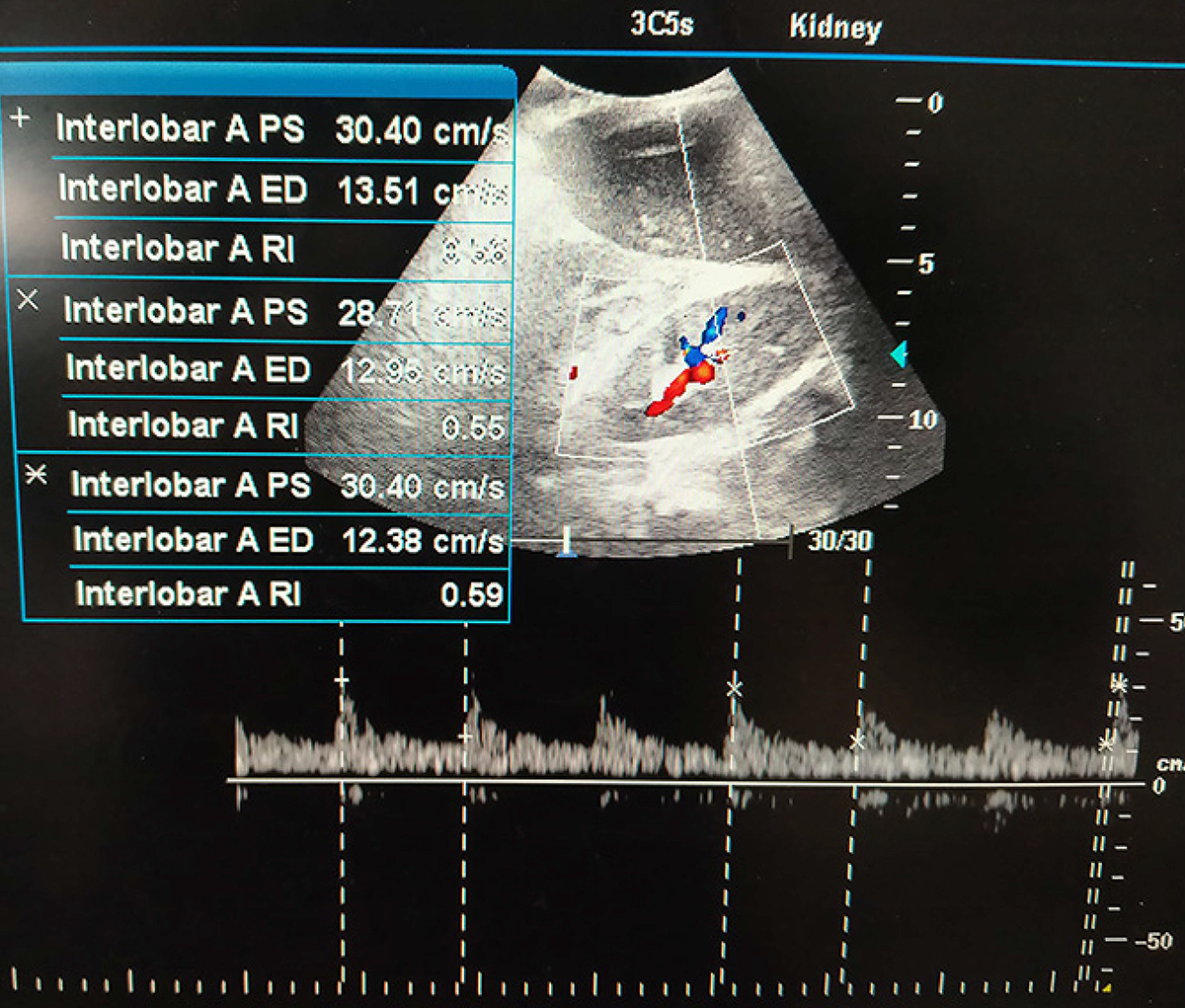

Supplement: Supplementary file 1 [file diagnostics-16-01153-s001.zip › Figure S2.tif]

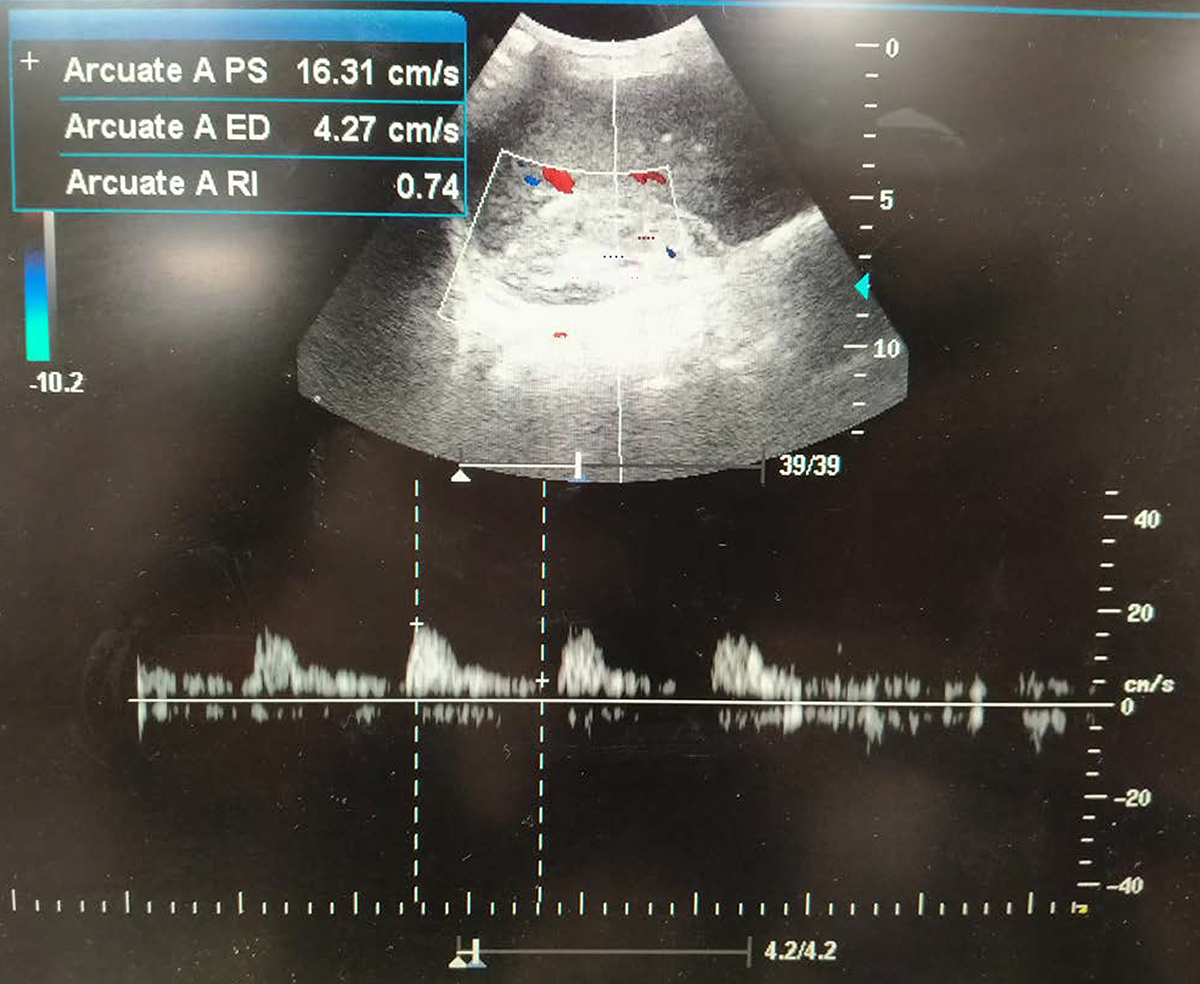

Supplement: Supplementary file 1 [file diagnostics-16-01153-s001.zip › Figure S3.tif]

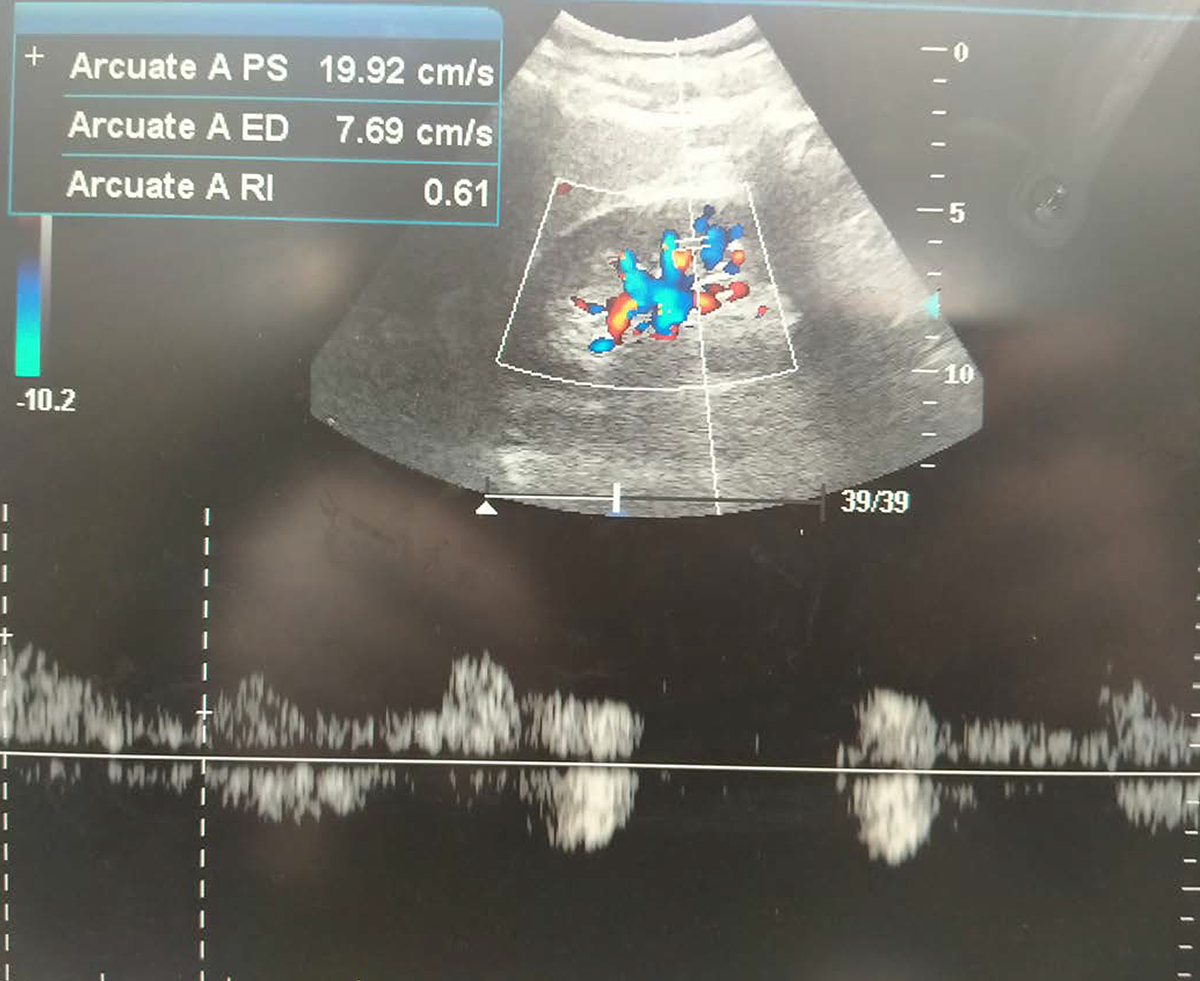

Supplement: Supplementary file 1 [file diagnostics-16-01153-s001.zip › Figure S4.tif]

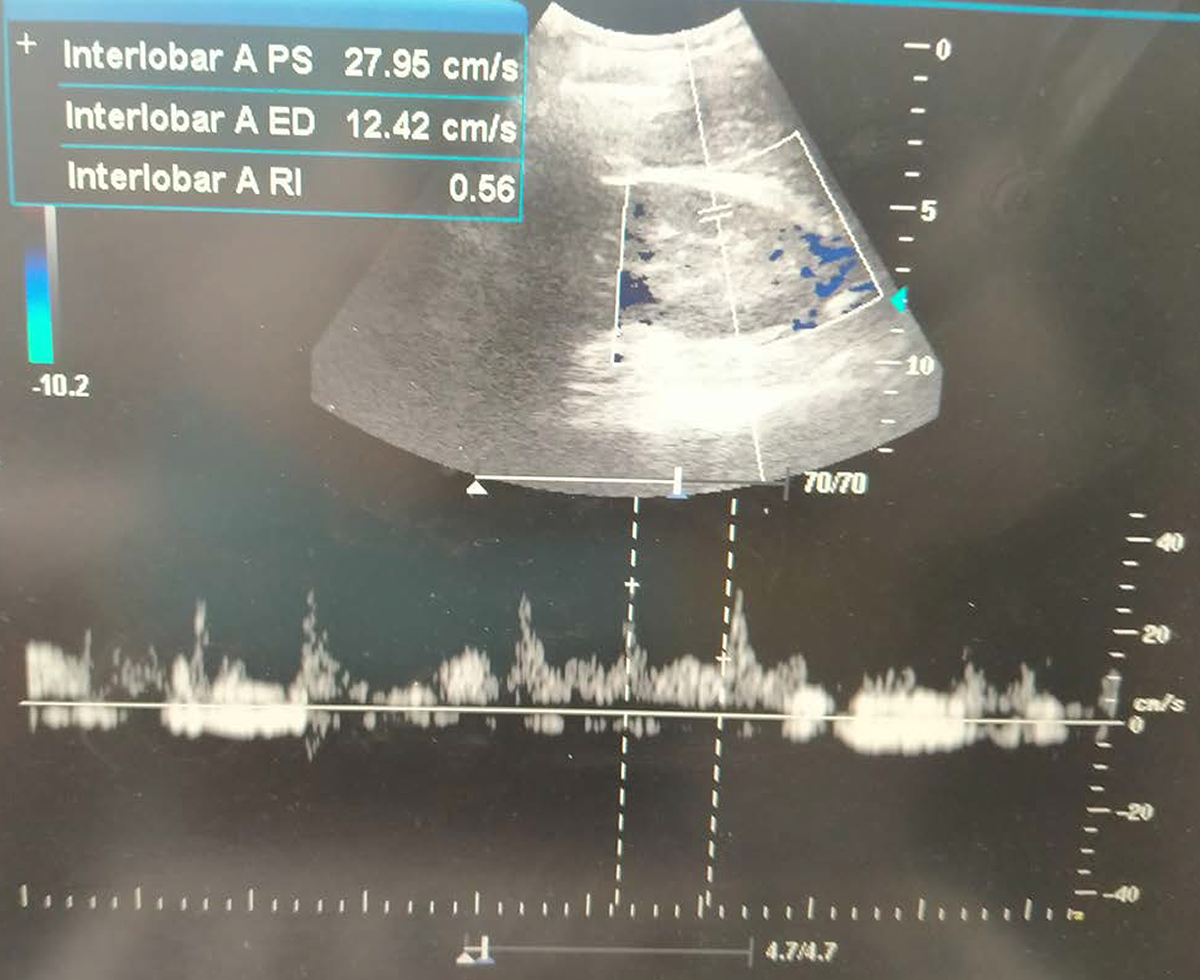

Supplement: Supplementary file 1 [file diagnostics-16-01153-s001.zip › Figure S5.tif]

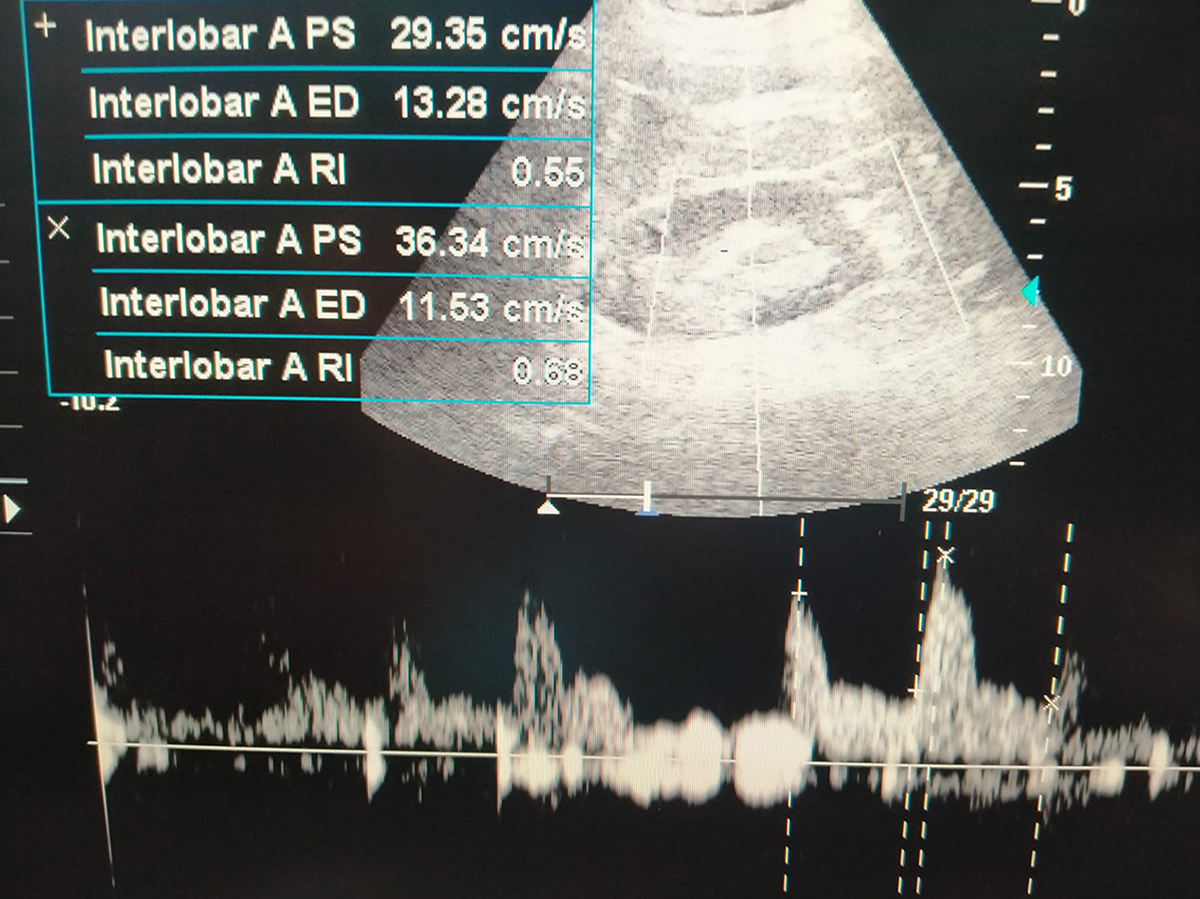

Supplement: Supplementary file 1 [file diagnostics-16-01153-s001.zip › Figure S6.tif]
